# Var2CSA DBL6-epsilon domain expressed in HEK293 induces limited cross-reactive and blocking antibodies to CSA binding parasites

**DOI:** 10.1186/1475-2875-7-170

**Published:** 2008-09-04

**Authors:** Pablo Fernandez, Nicola KViebig, Sébastien Dechavanne, Catherine Lépolard, Jürg Gysin, Artur Scherf, Benoit Gamain

**Affiliations:** 1 Institut Pasteur, Unité de Biologie des Interactions Hôte-Parasite, CNRS URA2581, Batiment Nicolle, 25, rue du Docteur Roux, F-75724 Paris Cedex 15, France; 2 Unité de Parasitologie Expérimentale, Université de la Méditerranée, Marseille, France; 3 Host Pathogen Interactions Unit, Institut Pasteur, Abymes, France

## Abstract

**Background:**

Pregnancy-associated malaria (PAM) is a serious consequence of *Plasmodium falciparum*-infected erythrocytes sequestration in the placenta through the adhesion to the placental receptor chondroitin sulfate A (CSA). Although women become resistant to PAM as they acquire transcending inhibitory immunity against CSA-binding parasites, hundreds of thousands of lives could be saved if a prophylactic vaccine targeting the surface proteins of placental parasites could be designed. Recent works point to the variant protein var2CSA as the key target for the development of a pregnancy-associated malaria vaccine. However, designing such a prophylactic vaccine has been hindered by the difficulty in identifying regions of var2CSA that could elicit broadly neutralizing and adhesion-blocking antibodies.

**Methods:**

Var2CSA is a very large protein with an estimated molecular weight of 350 kDa, and can be divided into six cysteine rich Duffy binding-like domains (DBL). The human embryonic kidney 293 cell line (HEK293) was used to produce secreted soluble recombinant forms of var2CSA DBL domains. The *Escherichia coli *expression system was also assessed for the domains not expressed or expressed in low amount in the HEK293 system. To investigate whether var2CSA binding DBL domains can induce biologically active antibodies recognizing the native var2CSA and blocking the interaction, mice were immunized with the refolded DBL3-X or the HEK293 secreted DBL6-ε domains.

**Results:**

Using the HEK293 expression system, DBL1-X, DBL4-ε and DBL6-ε were produced at relatively high levels in the culture supernatant, while DBL3-X and DBL5-ε were produced at much lower levels. DBL2-X and DBL3-X domains were obtained after refolding of the inclusion bodies produced in *E. coli*. Importantly, mice antisera raised against the recombinant DBL6-ε domain, specifically reacted against the surface of CSA-binding parasites and revealed adhesion blocking activity.

**Conclusion:**

This is the first report showing inhibitory binding antibodies obtained through a var2CSA recombinant DBL domain immunization protocol. These results support the current strategies using var2CSA as immunogen in the aim of blocking placental sequestration of malaria parasites. This work is a step towards the development of a var2CSA based vaccine that will prevent pregnancy-associated malaria and improve pregnancy outcomes.

## Background

Pregnancy-associated malaria (PAM) has serious adverse outcomes such as low birth weight neonates, increased perinatal and maternal mortality, anaemia and increased risk of hypertension in first-time pregnant mothers [1,2]. PAM is coupled with massive accumulation of parasitized erythrocytes (PEs) and monocytes in the placental intervillous blood spaces [3,4]. The basis for this accumulation in the placenta results from the capacity of placental PEs to bind to chondroitin sulfate A (CSA) but not to CD36, a common receptor for PEs sequestration in the microvasculature [5]. In endemic areas, women acquire antibodies against placental parasites over successive pregnancies, as they become resistant to PAM [6]. Women who have acquired antibodies against placental PEs have higher haemoglobin levels, deliver heavier babies and are much less susceptible to PAM than primigravid and HIV-infected women lacking these antibodies [7-9]. Furthermore, naturally acquired antibodies from multigravid women react against placental PEs or CSA-binding parasites collected around the world, indicating that target epitopes are globally conserved [6,10-12].

Recent evidences suggest that var2CSA, a member of the *Plasmodium falciparum *Erythrocyte Membrane Protein 1 (PfEMP1) family, may have an important role in PAM disease and immunity [13]. PfEMP1 proteins are clonally variant parasite adhesion ligands expressed on the surface of infected erythrocytes [14,15]. Var2CSA is a very large protein with an estimated molecular weight of 350 kDa, and can be divided into six Duffy binding-like domains (DBL1-6). Among them DBL2-X, DBL3-X and DBL6-ε specifically bind to CSA [16]. *Var2csa *gene orthologs are present in all parasite isolates [17] and are transcriptionally upregulated in both placental isolates and laboratory parasites selected to bind CSA [18-20]. Importantly, *var2csa *knock-out parasites revealed that no other parasite ligand can promote massive adhesion in the placenta [21-23]. Furthermore, the var2CSA protein is the target of naturally acquired maternal antibodies and the presence of var2CSA specific IgG has been correlated with higher birth weight babies [24-26].

All these data point to var2CSA as the key target for the development of a PAM vaccine, but a number of obstacles need to be overcome, such as the identification of regions in the large polymorphic molecule (350 kDa) able to induce broadly transcendent neutralizing antibodies that would de-sequester and/or mediate parasite phagocytosis. Given the var2CSA protein size, strategies to express the entire protein are not envisaged, but expression of correctly folded and biologically active cysteine-rich DBL domains is the most promising strategy.

In this study, an expression system was developed to produce recombinant DBL domains in the culture supernatant of transiently transfected Human Embryonic Kidney 293 cell line (HEK293) grown in serum-free medium in suspension. Using the HEK293 cell expression system, five DBL domains out of six were successfully secreted in the growth medium. As DBL2-X and DBL3-X were not found or found in low amount in the HEK293 growth medium, their expression in *Escherichia coli *was evaluated. Previously described CSA binding domains DBL3-X and DBL6-ε were then chosen to raise antisera in mice in order to evaluate their capacity to induce antibodies recognizing the PEs surface of different CSA-binding strains and for their capacity to inhibit FCR3-CSA PEs binding to Sc1D cells in static adhesion experiments.

## Methods

### HEK293 DBL expression and purification

*Var2csa *synthetic genes were designed with optimized codons for human expression. Synthetic genes encoding FCR3 var2CSA DBL domains (accession AY372123); DBL1-X (residues 58–383), DBL2-X (residues 530–863), DBL3-X (residues 1221–1548), DBL4-ε (residues 1594–1888), DBL5-ε (residues 2003–2270) and DBL6-ε (residues 2322–2590) were cloned into the pTT3 vector between the EcoRI and BamHI restriction sites. Synthetic genes contained an N-terminal murine Ig κ-chain leader sequence to allow secretion of the proteins [27] and a His6-tag on the C-terminus. Potential N-glycosylation sites were removed from synthetic genes by converting asparagine to glutamine or by replacing asparagine with an amino acid from another *var2csa *allele (Table 1).

**Table 1:** N-Glycosylation sites mutated in HEK293 expressed DBL domains

Domains	Boundaries	N-Glycosylation sites mutated*	
DBL1-x	58 – 383	5	**N**85**I**
			**N**147**Q**
			**N**356**K**
			**N**362**H**
			**N**380**Q**

DBL2-x	530 – 863	2	**N**692**Q**
			**N**730**Q**

DBL3-x	1221 – 1548	3	**N**1222**S**
			**N**1290**Q**
			**N**1428**K**

DBL4-ε	1594 – 1888	4	**N**1674**H**
			**N**1744**Q**
			**N**1749**Q**
			**N**1844**Q**

DBL5-ε	2003 – 2270	3	**N**2134**Q**
			**N**2210**Q**
			**N**2223**Q**

DBL6-ε	2322 – 2590	2	**N**2442**Q**
			**N**2537**Q**

*Asparagine residues were changed to glutamine or, in some instances, to amino acids found in other *var2csa *alleles at this location.

FreeStyle 293-F cells (Invitrogen) were grown in Freestyle 293 serum free expression medium and transfected with the pTT3 vector containing the synthetic genes following Invitrogen's recommendations. 72 hours post-transfection, cells were centrifuged and the culture medium was harvested. After filtration on a 0.22 μm filter, supernatants were concentrated five times using a 10 kDa cut-off Vivaflow 200 System (Vivasciences). Samples were then diafiltrated against PBS pH 7.2 (GIBCO) supplemented to 500 mM NaCl final concentration and charged on a HisTrap FF Ni-affinity column (GE Healthcare) previously equilibrated with the same buffer, and connected to an FPLC Akta System (Amersham Pharmacia Biotech). After washing, proteins were eluted with an imidazole gradient (0 to 0.5 M). Aliquots containing purified DBL domains were pooled and dialyzed against 0.9% NaCl. Purified proteins were subsequently concentrated using Macro- and Micro-sep concentrators (Pall/Gellman). Protein concentrations were determined using the Bio-Rad protein assay. Purity of the samples was checked by SDS-PAGE and Western blot.

### Prokaryotic expression, refolding and purification

Synthetic genes encoding for var2CSA DBL2-X and DBL3-X were designed with optimized codons for *E. coli *expression. Synthetic genes encoding FCR3 var2CSA (accession AY372123) DBL2-X (residues 530–863) and DBL3-X (residues 1221–1548) domains fused to a His6-tag on the C-terminus were cloned into the pET24a expression vector between the NdeI and EcoRI restriction sites. Transformed *E. coli *BL21 (DE3) cells were grown at 37°C in LB medium with 30 μg/ml of kanamycin to an absorbance of 0.5 at 600 nm, and were then induced with 1.0 mM isopropyl-β-D-thiogalactopyranoside (IPTG) for 3 h at 37°C under good aeration. Cells were harvested by centrifugation at 6,000 g. The pellets from 800 ml of culture were resuspended in 30 ml of 50 mM Tris.HCl, pH 8.5, containing 150 mM NaCl, in the presence of protease inhibitors. The cells were disrupted by sonication on ice and the suspensions were centrifuged for 20 min at 5,000 g. Pellets were resuspended and washed twice with 30 ml of 50 mM Tris.HCl, pH 8.5, containing 150 mM NaCl, in the presence of protease inhibitors, and finally centrifuged for 20 min at 10,000 g. The pellets containing DBL2-X or DBL3-X inclusion bodies were then denatured overnight at 25°C under agitation in 50 mM Tris.HCl pH 8.5 buffer, containing 150 mM NaCl, 8 M Urea and 5 mM Dithiothreitol (DTT). The suspensions were centrifuged for 30 min at 15,000 g and the pellet was discarded. Refolding was assayed with the AthenaES™ kit (Athena Environmental Sciences, Inc.) according to the manufacturer's instructions. Once the best condition was established, denatured proteins were immobilized on a HisTrap FF Ni-affinity column previously equilibrated with the same buffer, and connected to an FPLC Akta System. The proteins were refolded on the column by adding quickly the refolding buffer (50 mM Tris.HCl pH 8.5, 20 mM NaCl, 0.4 mM KCl, 0.5% Triton-X-100 and 0.5 mM DTT). After extensive washing with the refolding buffer without Triton-X-100, the protein was eluted with an imidazole gradient (0 to 0.5 M) and aliquots containing purified DBL domains were pooled. After dialysis against 0.9% NaCl and concentration by means of Macro- and Micro-sep concentrators, a further stage of gel filtration (Superdex 75, Amersham Pharmacia Biotech) was required to separate the monomeric proteins from the aggregated material and other impurities. Purified DBLs were subsequently concentrated by means of Macro- and Micro-sep concentrators. Protein concentration was determined using the Bio-Rad protein assay. Purity of the samples was checked by SDS-PAGE and Western blot.

Free thiol content was estimated using the Ellman's Reagent (Pierce) by comparison to a cysteine standard curve composed of known concentrations of Cysteine Hydrochloride Monohydrate following Pierce's recommendations.

### Parasite and cell culture

The *P. falciparum *FCR3, HB3 and 7G8 strains were maintained in culture according to standard conditions in O+ human erythrocytes in RPMI 1640 containing L-glutamine (Invitrogen) supplemented with 5% Albumax I, 1× hypoxanthine and 20 μg/ml gentamicin [28]. To maintain knob-positive parasites, cultures were routinely selected by gelatin flotation using Plasmion (Fresenius Kabi) [29]. Parasites were tested *Mycoplasma *negative by PCR. Laboratory isolates, FCR3, 7G8 and HB3 were initially selected on bovine CSA or on the human choriocarcinoma placenta BeWo cell line and subsequently maintained by panning to the BeWo cell line as previously described [30]. CD36 or CSA binding phenotypes of PEs were verified on receptors immobilized on plastic Petri dishes as previously described [30].

### Balb/c immunization

Two groups of three Balb/c mice (Charles River) received a primary subcutaneous injection of 20 μg refolded recombinant DBL3-X or secreted DBL6-ε protein dissolved in 100 μl of NaCl 0.9% and emulsified at 1:1 in 100 μl of complete Freund adjuvant (Pierce). Two additional injections were performed at 4 week intervals by using the same amount and antigen batches emulsified at 1:1 in incomplete Freund adjuvant. Mice were bled by orbital sinus puncture one day before the primary antigen injection and each time preceding an antigen boost, and 2, 4 and 6 weeks after the 3rd and last boost. Serum samples were collected after centrifugation of the blood, de-complemented for 30 min at 56°C and stored at -20°C until use.

### Flow cytometry

Synchronous PEs cultures (3–12% parasitaemia) at mid/late trophozoite stages were purified using the VarioMACS and CS columns (Miltenyi) as previously described [31] and resuspended to 10^7 ^PEs/ml in PBS 0.2% BSA. PEs were added to 96-well U-bottom plates and the mouse serum (heat inactivated and pre-adsorbed on uninfected erythocytes from mock culture) was added into the wells to get a 1:20 dilution of serum to cell suspension (using a final volume of 50 to 100 μl). After incubation at room temperature for 30 min, 100 μl of PBS containing 0.2% BSA were added, and cells were centrifuged at 2000 rpm for 2 min. Cells were washed twice with 160 μl of PBS/0.2% BSA and resuspended in 50 μl PBS/0.2% BSA containing the secondary antibody (anti-mouse IgG Alexa 488) diluted 1:100. After incubation on ice in the dark for 30 min, cells were centrifuged and washed as before, and resuspended in 2–4% paraformaldehyde in PBS for fixation overnight at 4°C. Fixed cells were centrifuged at 2000 rpm for 2 min and resuspended in 100 μl PBS before transfer to flow cytometry tubes. Analysis was carried out on a FACScan using CellQuest software (BD Biosciences). The flow cytometer gated the PEs' population based on forward scatter and side scatter, and cells were plotted as a histogram by fluorescence in channel 1 (Alexa fluor^®^488). At least 10000 cells were counted for each sample. The level of fluorescence was stated as the Geometric Mean Fluorescence Index (MFI) of all the gated cells as determined in at least two independent experiments. Data were then converted to normalized MFI by subtracting the preimmune MFI value from the immune MFI value.

### Inhibition experiments

Cytoadhesion microassays were performed on confluent *Saimiri *brain endothelial cell (SBEC) Sc1D cells as previously described [32]. Briefly, endothelial cells were rinsed twice with cytoadhesion medium at pH 7.2 before the addition to the cells of 20 μl of serum (diluted 1:5) preincubated with 20 μl of PEs (at 10^7 ^cells/ml). The slides were incubated for two hours at 37°C in a CO_2 _incubator. Non-adherent PEs were removed by washing with cytoadhesion medium and cytoadherent PEs were counted under a light microscope (Nikon TMS, magnification of ×300), in four randomly selected fields (area of 0.2827 mm^2^) for each spot.

## Results

### Expression and Purification of recombinant DBL domains

Var2CSA is a key target for the development of a PAM vaccine. Due to the large protein size, the most promising strategy is a vaccine based on var2CSA protein subunits. Therefore, the six var2CSA DBL domains from the FCR3 strain were tested for expression as secreted His-tagged recombinant proteins in the human embryonic cell line HEK293. For that purpose synthetic genes were designed with optimized codons for human expression and with an N-terminal murine Ig κ-chain leader sequence to allow secretion of the proteins and a His6-tag on the C-terminus. As *P. falciparum *proteins are not N-glycosylated, potential N-glycosylation sites were removed from synthetic genes by converting asparagine to glutamine or by replacing asparagine with an amino acid from another *var2csa *allele (Table 1).

**Figure 1:**
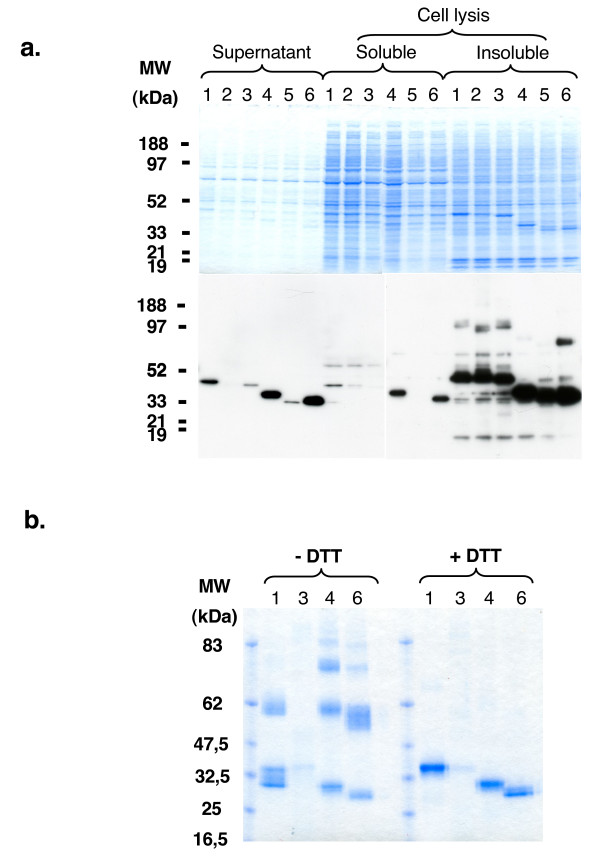
**Protein purification of FCR3 var2CSA DBL domains expressed in HEK293**. a. SDS PAGE and Western blot analysis. NuPAGE Novex 4–12% Bis-Tris gels under reduced conditions were loaded with HEK293 extracts obtained after transfection with the different FCR3 var2CSA DBL domains. Gels were either stained with Coomasie blue or transferred to a PVDF membrane to detect recombinant products using an anti-His Tag antibodies. Lanes 1, 2, 3, 4, 5, and 6 correspond respectively to DBL1-X, DBL2-X, DBL3-X, DBL4-ε, DBL5-ε, and DBL6-ε. Culture supernatant, soluble and insoluble fractions after cell lysis are indicated. b. Electrophoresis of purified HEK293 expressed DBL domains. NuPAGE Novex 4–12% Bis-Tris gels under reduced (+DTT) or non reduced (-DTT) conditions were loaded with DBL1-X, DBL3-X, DBL4-ε and DBL6-ε obtained after His-Tag purification and stained with Coomasie blue. Lanes 1, 3, 4, and 6 correspond respectively to DBL1-X, DBL3-X, DBL4-ε and DBL6-ε domains. Protein yields were 0.6 mg/L for DBL1-X, 0.9 mg/l for DBL4-ε and 5 mg/l for DBL6-ε.

Using this expression system, DBL1-X, DBL4-ε and DBL6-ε were produced at relatively high levels in the culture supernatant, while DBL3-X and DBL5-ε were produced at much lower levels (Figure 1a). No expression of DBL2-X could be detected in the culture supernatants. However, after cell lysis, all the recombinant proteins were found in the insoluble fraction, indicating that some of the proteins are likely to be retained in the cells due to their incorrect conformation.

After purification on a HisTrap FF Ni-affinity column, highly pure recombinant (over 95%), DBL1-X, DBL4-ε and DBL6-ε were obtained at a yield ranging from 0.6 to 5 mg/l of cell culture supernatant, while only a minimal amount of DBL3-X was obtained (Figure 1b). Under reducing conditions in SDS-PAGE, all four proteins migrated at the expected molecular weight (Figure 1b). DBL4-ε and DBL6-ε migrated as a double-band as a consequence of the presence of two isoforms of the protein, which could result from post-transductional modifications such as O-glycosylation. N-terminal sequencing and SDS-PAGE indicated that proteins remained intact, without proteolytic degradation occurring during the purification procedures.

As the chosen strategy was to express CSA-binding domains rather than non CSA binding domains, DBL2-X and DBL3-X domains were tested for expression in *E. coli*. For that purpose, synthetic genes encoding for var2CSA DBL2-X and DBL3-X fused to a His6-tag on the C-terminus were designed with optimized codons for *E. coli *expression.

**Figure 2:**
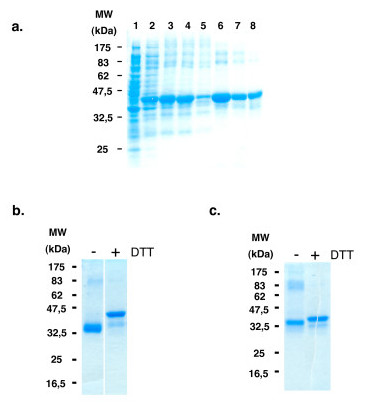
**DBL2-X and DBL3-X expression in *E. coli *and purification**. a. Electrophoresis in an SDS 12% polyacrylamide gel of aliquots from different steps in expression, refolding and purification of DBL3-X. Lane 1, non-induced BL21 (DE3) (containing pET24a-DBL3-X plasmid); lane 2, induced BL21 (DE3) containing the pET24a-DBL3-X plasmid; lane 3, inclusion bodies suspension; lane 4, inclusion bodies after washing and denaturation in Urea 8 M; lane 5, affinity column flow through; lane 6, portion of DBL3-X lost during refolding onto the affinity column; lane 7, affinity column elution pool; lane 8, molecular size exclusion column purified DBL3-X. b. Electrophoresis of refolded DBL3-X in a SDS 12% polyacrylamide gel before (-) and after (+) reduction with dithiothreitol (DTT). c. Electrophoresis of refolded DBL2-X in a SDS 12% polyacrylamide gel before (-) and after (+) reduction with dithiothreitol (DTT). Higher mobility before reduction indicates the presence of disulfide linkages.

Recombinant DBL2-X and DBL3-X expressed in *E. coli *accumulate in inclusion bodies as insoluble, misfolded aggregates. Misfolded DBL domains were solubilized in 8 M urea, purified under denaturing conditions by metal-affinity chromatography and refolding tests were carried out by the method of rapid dilution. Using that strategy, a buffer containing 0.5% Triton-X-100 was identified as the best solution for refolding both insoluble recombinant DBL domains. However, due to the incapacity to completely remove Triton X-100 through classical methods, such as dialysis or filtration, the refolding process was done after immobilization of the denatured proteins on a metal-affinity column. In this procedure, DBL domains solubilized in 8 M urea were immobilized onto the metal-affinity column and the denaturation solution changed rapidly for the previously identified refolding solution containing Triton-X-100 (Figure 2a). Although due to sudden buffer change some DBL detached from the column, an important fraction of the protein was retained on the column. After extensive washing with the refolding buffer without the detergent, the proteins were eluted using an imidazole gradient (Figure 2a). After dialysis against 0.9% NaCl, gel filtration chromatography using Superdex 75 was performed to purify highly pure (over 95%) recombinant DBL monomers to homogeneity (Figure 2a). Using that strategy, the protein yield after refolding and all purification steps was 4 mg/l of cell culture for DBL3-X (Figure 2b) and 0.3 mg/l of cell culture for DBL2-X (Figure 2c). Due to the low yield recovered, DBL2-X was not further characterized. However, refolded and purified DBL3-X was characterized using a variety of biochemical and biophysical methods. N-terminal sequencing of recombinant DBL3-X yields the expected sequence, namely, MNATN. No other sequence was detected. Refolded DBL3-X migrated slower on SDS-PAGE gels after reduction with DTT indicating the presence of disulfide bonds (Figure 2b). Free thiol content was estimated by the method of Ellman to further examine the oxidation state of refolded DBL3-X. Free thiols can be clearly detected up to 30 μM thiol concentrations in this assay. No free thiols are detected in refolded DBL3-X at a protein concentration of 100 μM. Given that DBL3-X contains 12 cysteines, greater than 96% of cysteines are thus disulfide linked.

### Antibodies to var2CSA DBL6-ε partially cross-react with different CSA binding strains

**Figure 3:**
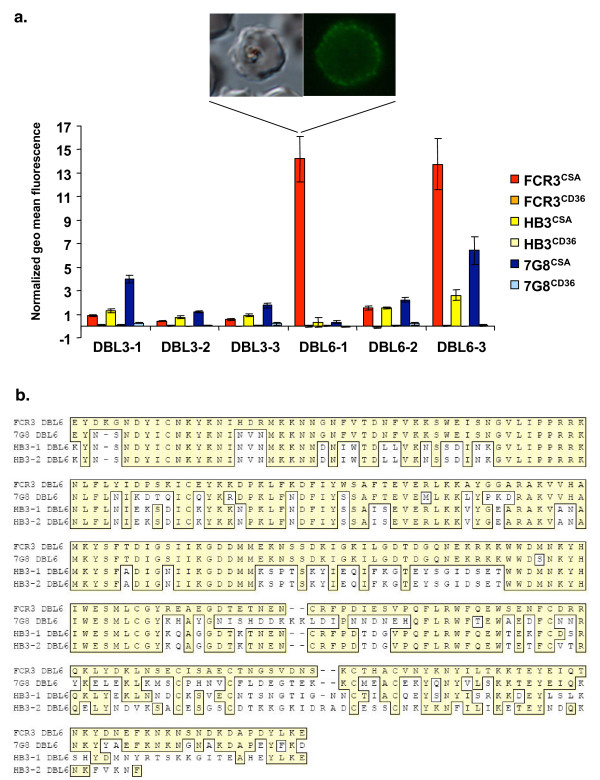
**PEs surface recognition**. a. Anti-var2CSA DBL mouse sera were tested for surface recognition of erythrocytes infected with parasites of the FCR3, 7G8 and HB3 strains with CSA- and CD36 binding phenotypes, respectively. Analysis was performed using flow cytometry and fluorescence microscopy. A representative example of a specific surface immunolabeling using the DBL6-1 antisera and the corresponding differential interferential contrast microscopy field are shown. Flow cytometry data shown are the normalized geometric mean fluorescence index (MFI) (± SD) obtained by subtracting the preimmune MFI value from the immune MFI value. b. Alignment of the DBL6-ε var2CSA sequences from the 3 parasite lines used to assess the antisera cross-reactivity by FACS. Conserved residues between FCR3 sequence and the other sequences are boxed with a yellow background.

To investigate whether the recombinant var2CSA DBL domains can induce biologically active antibodies recognizing the native var2CSA, mice were immunized with the refolded DBL3 or the HEK293 secreted DBL6-ε. Final bleed antisera were evaluated for their capacity to recognize the surface of FCR3^CSA ^binding PEs by liquid immunofluorescence and flow cytometry (Figure 3a). Two out of the three mice immunized with DBL6-ε (DBL6-1 and DBL6-3) had high level of antibodies recognizing the surface of FCR3^CSA ^PEs. Very low reactivity was detected for mouse DBL6-2. However, in contrast to DBL6-ε, the DBL3-X antisera did not react or reacted very weakly with the surface of FCR3^CSA ^PEs (Figure 3a).

To investigate whether mice antisera could also recognize other var2CSA variants, flow cytometry analysis was performed on two other CSA-binding parasite lines from South America (7G8^CSA^) and Central America (HB3^CSA^) (Figure 3b). Two out of the three mice immunized with DBL6-ε (DBL6-2 and DBL6-3) had a significant level of antibodies recognizing the surface of 7G8^CSA ^PEs and a low amount of antibodies reacting against HB3^CSA ^(Figure 3a). Curiously all three DBL3-X immunized antisera reacted against 7G8^CSA ^PEs and recognized weakly the HB3^CSA ^strain, even if they did not recognize the surface of the homologous strain FCR3. No significant recognition of all the antisera was observed against the CD36 binding parasites.

### Var2CSA DBL6-ε antibodies partially inhibit CSA adhesion

**Figure 4:**
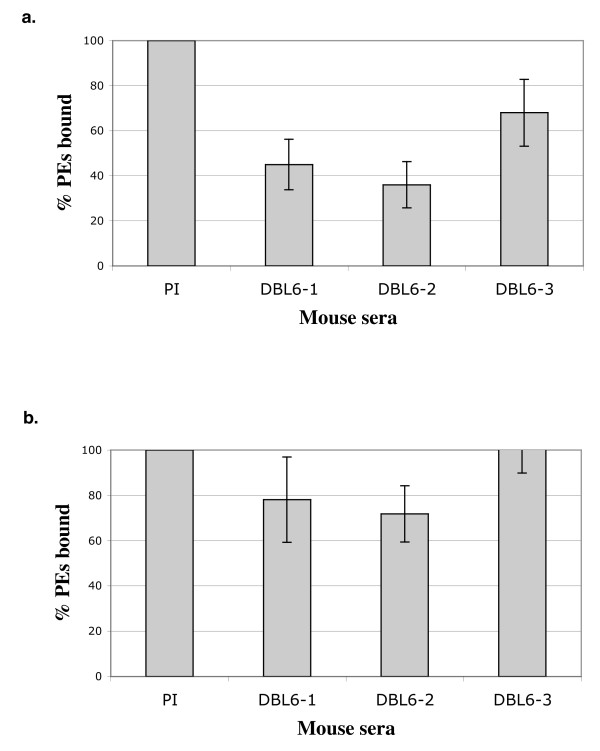
**Inhibition of PEs adhesion**. Mouse antisera (1:10 dilution) were tested for inhibition of binding of PEs to Sc1D cells in static binding assays at D85 after immunization (a) and for final bleeds (b). The number of bound PEs in the presence of preimmune serum (PI) was used as a reference (100%). The numbers of bound PEs for the three anti-DBL6-ε sera were then expressed as a percentage relative to the PI reference value. Values represent the means of two individual experiments performed in duplicates.

In order to evaluate the inhibitory capacities of the DBL3-X and DBL6-ε antisera, the binding of FCR3^CSA ^PEs to the CSA expressing endothelial cell line Sc1D was assessed in the presence of non-immune or immune antisera at a final 1:10 dilution. One month after the third injection (Day 85), the three DBL6-ε immunized mice recognized the surface of FCR3^CSA ^binding PEs by liquid immunofluorescence and inhibited PEs cytoadhesion to Sc1D cells from 32 to 64% (Figure 4a). However, no inhibition was observed using the DBL3-X antisera. After an additional boost, DBL6-ε immunized mice were sacrificed and the final bleeds were tested for inhibition. Although the final DBL6-ε antisera bleeds recognize the surface of CSA-binding PEs (Figure 3a), almost no inhibitory activity could be detected (Figure 4b), indicating a change in the immune response. From these data we can conclude that the DBL6-ε domain can induce an immune response that block PEs adhesion, indicating that it is a critical target domain.

## Discussion

Several lines of evidence point to var2CSA as the leading vaccine candidate to prevent PAM. However, the major limiting steps for validating this molecule as a protective immunogen reside in its high molecular weight and the production of sufficient functional recombinant protein. Indeed, the var2CSA protein size (around 350 kDa) and the hydrophobic and cysteine-rich sequence contribute to aggregation as insoluble material upon expression in the available prokaryotic systems. Therefore we assessed the human embryonic kidney cell line HEK293 as a new system for expressing var2CSA DBL domains from the FCR3 strain as secreted His-tagged recombinant proteins. Engineering the expression vector with a sequence signal allowed us to obtain high quality soluble affinity purified material in a quick and easy way. Although the HEK293 expression system was useful for secreting in relatively high quantity (up to 5 mg/l) three out of six different domains in the culture supernatant, the three others were either absent or present in very low quantities in the culture supernatant. We also showed here that antisera from mice immunized with DBL6-ε recognized the surface of the homologous parasite FCR3^CSA ^and a variable degree of cross-reactivity was observed for 7G8^CSA ^and HB3^CSA ^parasites in individual mice. As 7G8 var2CSA is closer to FCR3 var2CSA than both var2CSA variants present in HB3 (HB3.1 and HB3.2) (Figure 3b), this could explain the higher cross-reactivity observed against the 7G8^CSA ^PEs surface. Considering that DBL6-ε domain is the least conserved var2CSA DBL domain, this limited cross-reactivity is somehow expected. It also suggests that the immunodominant epitopes are in the polymorphic region, whereas the conserved block may not be exposed in the native var2CSA molecule (Figure 3b).

As two of the previously described CSA binding domains present in var2CSA (DBL2-X and DBL3-X) were not expressed or expressed in low amount in the growth supernatant, the *E. coli *expression system was evaluated. The proteins were recovered from inclusion bodies under denaturing conditions and a column refolding based process allowed us to obtain DBL3-X refolded material. Surprisingly, antisera from mice immunized with DBL3-X did not react with the surface of the homologous CSA binding PEs, but recognized weakly the surface of 7G8^CSA ^PEs. Although DBL3-X is one of the most conserved var2CSA DBL domains, it is hard to understand why the antisera react in a better way with a heterologous antigen than with the homologous one. One explanation could be due to a better accessibility to the antigen on the surface of 7G8^CSA ^PEs compared to FCR3^CSA ^or HB3^CSA ^parasites, or that the DBL3-X domain was not correctly refolded as it is in the context of the whole PfEMP1.

The DBL3-X and DBL6-ε immunized mice immunological responses were also followed by testing the antisera for their ability in blocking PEs adhesion to Sc1D cells. We showed here that all the antisera raised against DBL6-ε inhibited PEs adhesion with values ranging from 32% to 64% inhibition at D85 after immunization. However, after an additional boost and even with a good surface reactivity, the inhibitory activity was strongly reduced in the final bleed, indicating that other epitopes are targeted by these antisera. It is therefore conceivable that most anti-DBL6-ε antibodies are against the surface of the CSA binding PEs but do not inhibit adhesion. Another explanation could be that some of these non-inhibitory antibodies could compete with the inhibitory antibodies preventing the latter from reaching the CSA binding pocket. In terms of vaccine development, as it seems that the balance between inhibitory and non-inhibitory antibodies may change during the course of immunization, it may be important to specifically target the critical regions involved in adhesion in order to overcome this potential problem.

## Conclusion

This is the first report showing adhesion inhibitory antibodies obtained through a var2CSA recombinant DBL domain immunization protocol. These results support the current strategies using var2CSA as immunogen in the aim of blocking placental sequestration of malaria parasites and set up the basis for developing a vaccine against pregnancy-associated malaria based on this protein.

## List of abbreviations

PEs: Parasitized Erythrocytes; CSA: Chondroitin Sulfate A; PAM: Pregnancy Associated Malaria; PfEMP1: *P. falciparum *Erythrocyte Membrane Protein 1; DBL: Duffy binding-like; HEK293: Human Embryonic Kidney 293; SBEC: Saimiri brain endothelial cell.

## Competing interests

The authors declare that they have no competing interests.

## Authors' contributions

PF participated in the design of the study, carried out the expression and purification of the recombinant DBL domains and wrote the manuscript. NV participated in the design of the study, performed the FACS experiments and wrote the manuscript. SD, CL and JG evaluated the inhibitory capacities and the surface reactivity by liquid IFA of the antisera. AS participated in the design of the study and wrote the manuscript. BG expressed DBL domains, conceived the study, participated in its design and coordination and wrote the manuscript. All authors read and approved the final manuscript.
